# Delayed autonomic neuropathy in a patient with diethylene glycol poisoning: a case report

**DOI:** 10.1002/ams2.267

**Published:** 2017-03-27

**Authors:** Hiroki Kamada, Hideaki Suzuki, Saori Yamamoto, Ryosuke Nomura, Shigeki Kushimoto

**Affiliations:** ^1^ Division of Emergency and Critical Care Medicine Tohoku University Graduate School of Medicine Sendai Japan; ^2^ Department of Cardiovascular Medicine Tohoku University Graduate School of Medicine Sendai Japan

**Keywords:** Acetamiprid, autonomic neuropathy, delayed neuropathy, diethylene glycol, poisoning

## Abstract

**Case:**

A 72‐year‐old man presented to our hospital after ingesting insecticide containing approximately 2 mL/kg diethylene glycol, which exceeded the lethal dose of 1 mL/kg. The patient recovered from critical symptoms on acute phase until day 3, but received artificial ventilation for muscle weakness secondary to sensorimotor neuropathy on days 11–54.

**Outcome:**

Even after marked improvement from sensorimotor neuropathy, the patient continued to complain of orthostatic hypotension. Autonomic neuropathy was identified by positive result of a head‐up tilt test, and reduction in coefficient of variation of R‐R intervals and cardiac iodine‐123‐metaiodobenzylguanidine uptake for the assessment of cardiac sympathetic activity. The patient's symptoms fully recovered 2 years after the exposure to diethylene glycol.

**Conclusion:**

This case shows the first report of delayed autonomic neuropathy after recovery from severe sensorimotor neuropathy, and suggests the importance of continuous monitoring for late‐onset neurological complications.

## Introduction

Diethylene glycol (DEG) is widely used as an excellent solvent for water‐insoluble chemicals and drugs. Diethylene glycol intoxication is marked by gastrointestinal symptoms and hepatorenal injury in the acute phase and delayed neurological complications with evidence of peripheral demyelination that causes quadriparesis and ventilator dependence in severe cases.^1^, ^2^, ^3^, ^4^ However, autonomic neuropathy in DEG poisoning has never been reported. Here, we report a case of delayed autonomic neuropathy after recovery from severe sensorimotor neuropathy.

The report of this case was approved by the Ethics Committee of the Tohoku University Graduate School of Medicine (2015‐1‐668; Sendai, Japan).

## Case

A 72‐year‐old man was transferred to our hospital because he was suspected to have ingested insecticide as a suicide attempt. On admission, he was disoriented with a Glasgow Coma Scale score of 11 (E3V3M5) and suffered from nausea and vomiting. His blood pressure (BP), heart rate, respiratory rate, and body temperature were 87/45 mmHg, 76 b.p.m., 24 breaths/min, and 36.2°C, respectively. An atrioventricular block was observed by electrocardiogram on admission. Blood gas analysis revealed lactic acidosis with pH 7.422, pCO_2_ 22.4 mmHg, pO_2_ 126 mmHg, HCO_3_ 14.3 mmol/L, lactate 9.0 mmol/L, and anion gap 20.3 mmol/L. Renal replacement therapy was used on days 1–30 because of the progression of metabolic acidosis and acute kidney injury resulting in anuria. The patient was agitated and uncooperative, and required sedation under endotracheal intubation with ventilator support. Therefore, the patient was well oriented and had recovered from circulatory failure and lactic acidosis until day 3. However, the patient's consciousness was disturbed owing to carbon dioxide retention, and artificial ventilation was started on day 11. He became ventilator‐dependent status and could not respond to any commands after day 16. Although no remarkable finding was observed in brain computed tomography, magnetic resonance image, or electroencephalogram, a nerve conduction study on day 35 showed a reduction in the amplitudes and conduction velocities of compound muscle and sensory nerve action potentials in the limbs, indicating that his ventilator‐dependence and unresponsiveness were secondary to polyneuropathy. The patient's responsiveness to verbal stimuli was recovered on day 44, and artificial ventilation was withdrawn on day 54. Serum toxicological studies and scrupulous interviews revealed that the patient had ingested approximately 100 mL insecticide containing 2% acetamiprid, 97% DEG, and 1% surfactant, which was approximately equivalent to 2.06 mL/kg DEG.

**Figure 1:**
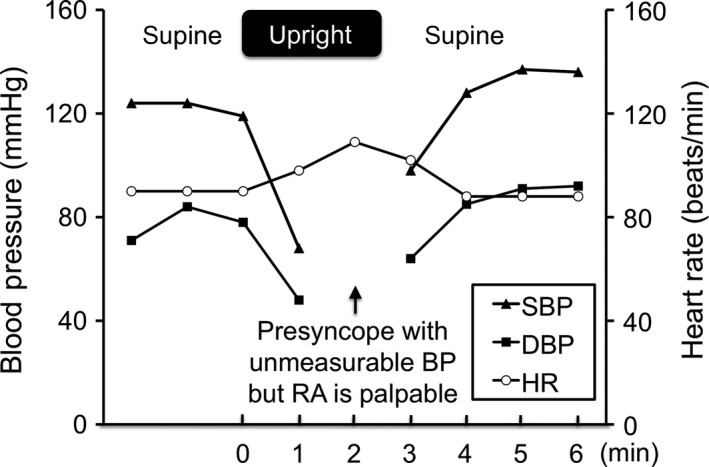
Results of a head‐up tilt test on day 70 in a 72‐year old man who ingested a lethal dose of diethylene glycol. His blood pressure (BP) drastically decreased within 1 min of standing in an upright position with slight increase in heart rate (HR). The patient suffered from presyncope with unmeasurable BP, although his radial artery (RA) was palpable. His BP recovered within a few minutes of returning to the supine position. DBP, diastolic blood pressure; SBP, systolic blood pressure.

**Figure 2:**
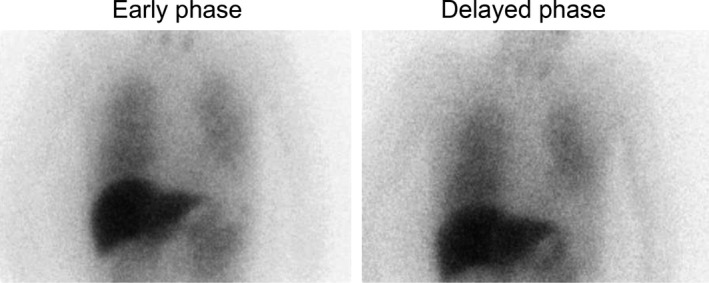
Results of iodine‐123‐metaiodobenzylguanidine scintigraphy on day 64 in a 72‐year‐old man who ingested and survived a lethal dose of diethylene glycol. Cardiac iodine‐123‐metaiodobenzylguanidine uptake was significantly reduced both in early and delayed phases.

After withdrawn from the ventilator, the patient became fully oriented, could speak frequently, and his muscle weakness of the limbs was improved. However, he complained of dizziness every time he tried to maintain a standing position for a certain amount of time during rehabilitation, although the muscle strength of his legs was adequate. The patient was subjected to a head‐up tilt test at 70° on day 70. The test was positive and finished in 2 min after tilting because the subject felt uncomfortable with unmeasurable BP (Fig. 1). His coefficient of variation of R‐R intervals was 1.04%. Iodine‐123‐metaiodobenzylguanidine (^123^I‐MIBG) scintigraphy showed a marked reduction in cardiac ^123^I‐MIBG uptake with heart/mediastinum ratio of 1.49 on early phase and 1.44 on delay phase without scatter correction (Fig. 2). These lines of evidence indicated that he suffered from autonomic neuropathy. We thus undertook his rehabilitation in a sitting position or raising his upper body on the bed. The change in his rehabilitation program motivated the patient to undergo rehabilitation. On day 85, he was discharged to rehabilitation hospital with the use of a wheelchair without assistance but could not keep a standing position. Although receiving mental health support from a psychiatric hospital, the patient had recovered from any neurological symptoms within 2 years of exposure to DEG.

## Discussion

This patient manifested various multiphase symptoms owing to toxicities from an insecticide containing acetamiprid and DEG. Patients with acute acetamiprid intoxication tend to recover within a few days without any complication, and there have been no reports of delayed polyneuropathy following acute intoxication.^5^, ^6^ However, the patient ingested approximately 2.06 mL/kg DEG, which exceeded the lethal dose of 1 mL/kg for adults.^1^ Although the precise mechanism of DEG poisoning has not been fully understood, 2‐hydroxyethoxyacetic acid, which is a metabolic product of DEG by oxidation, seems to be a major contributor to the neurological and renal toxidromes.^7^ In patients with DEG intoxication, delayed neuropathy can be anticipated at least 5–10 days after ingestion, including weakness in limbs and respiratory muscles, coma, and encephalopathy.^1^ Differential diagnosis for delayed neuropathy in this case included intensive care unit‐acquired weakness/critical illness polyneuropathy, uremic neuropathy, and deficiency of vitamin B and zinc. The patient was weaned from ventilatory support and was so active before reintubation on day 11 that intensive care unit‐acquired weakness/critical illness polyneuropathy could not have caused respiratory weakness. Although the patient received renal replacement therapy on days 1–30, he never showed uremia or its symptoms, except for metabolic acidosis on day 1. Blood tests showed normal values of vitamin B and zinc. Therefore, delayed polyneuropathy could not be explained by the differential diagnosis and might have been caused by DEG intoxication in this case.

This patient showed autonomic neuropathy which was characterized by orthostatic hypotension, positive result from the head‐up tilt test, and reduction in coefficient of variation of R‐R intervals and cardiac ^123^I‐MIBG uptake. To the best of our knowledge, however, this is the first report of autonomic neuropathy complicated with DEG intoxication. The reason why there has been no report of autonomic neuropathy in DEG intoxication is partly because few cases can survive from severe delayed neuropathy in DEG intoxication.^3^, ^4^ Rapid‐progressing fatal paralysis and brain atrophy, edema, and infarction, which might be irreversible, were also reported.^2^, ^8^, ^9^ Therefore, the high mortality rate of severe neurological complications might conceal an existence of autonomic neuropathy in DEG poisoning. One possible explanation for persistent autonomic neuropathy even after recovery of sensorimotor nerves is its high content rate of unmyelinated fibers. Unmyelinated axons and long or sparsely myelinated axons are more vulnerable to degeneration than thick myelinated axons.^10^ Therefore, degeneration of autonomic nerves might be more severe than that of sensorimotor nerves in this case. Management of the renal dysfunction due to DEG intoxication using renal replacement therapy might improve the severity and prevent the progression of delayed neuropathy as well as renal dysfunction, because DEG is water soluble, has a low molecular mass, and is dialyzable.^11^ In our case, renal replacement therapy might have prevented the development of irreversible renal dysfunction and DEG‐induced neuropathy. Finally, the patient completely recovered from any neurological symptoms, including autonomic neuropathy, 2 years after exposure to DEG.

## Conclusion

This case shows the first report of delayed autonomic neuropathy sustained after recovery from severe sensorimotor neuropathy in DEG intoxication, and suggests the importance of continuous monitoring for late‐onset neurological complications.

## Conflict of Interest

None declared.

## References

[1] Schep LJ , Slaughter RJ , Temple WA *et al* Diethylene glycol poisoning. Clin. Toxicol. 2009; 47: 525–35. 10.1080/15563650903086444 19586352

[2] Rollins YD , Filley CM , McNutt JT *et al* Fulminant ascending paralysis as a delayed sequela of diethylene glycol (Sterno) ingestion. Neurology 2002; 59: 1460–3. 12427908 10.1212/01.wnl.0000032498.65787.8d

[3] Hasbani MJ , Sansing LH , Perrone J *et al* Encephalopathy and peripheral neuropathy following diethylene glycol ingestion. Neurology 2005; 64: 1273–5. 15824363 10.1212/01.WNL.0000156804.07265.1A

[4] Conklin L , Sejvar JJ , Kieszak S *et al* Long‐term renal and neurologic outcomes among survivors of diethylene glycol poisoning. JAMA Intern. Med. 2014; 174: 912–7. 24819553 10.1001/jamainternmed.2014.344 PMC4547768

[5] Phua DH , Lin CC , Wu ML *et al* Neonicotinoid insecticides: an emerging cause of acute pesticide poisoning. Clin. Toxicol. (Phila.) 2009; 47: 336–41. 19514881 10.1080/15563650802644533

[6] Imamura T , Yanagawa Y , Nishikawa K *et al* Two cases of acute poisoning with acetamiprid in humans. Clin. Toxicol. (Phila.) 2010; 48: 851–3. 20969506 10.3109/15563650.2010.517207

[7] Sosa NR , Rodriguez GM , Schiner JG *et al* Clinical, laboratory, diagnostic, and histopathologic features of diethylene glycol poisoning–Panama, 2006. Ann. Emerg. Med. 2014; 64: 38–47. 24439712 10.1016/j.annemergmed.2013.12.011

[8] Alfred S , Coleman P , Harris D *et al* Delayed neurologic sequelae resulting from epidemic diethylene glycol poisoning. Clin. Toxicol. (Phila.) 2005; 43: 155–9. 15902788

[9] Imam YZ , Kamran S , Karim H *et al* Neurological manifestation of recreational fatal and near‐fatal diethylene glycol poisonings: case series and review of literature. Medicine (Baltimore) 2014; 93: 1–7. 10.1097/MD.0000000000000062 PMC4616334 25170933

[10] Orimo S , Uchihara T , Kanazawa T *et al* Unmyelinated axons are more vulnerable to degeneration than myelinated axons of the cardiac nerve in Parkinson's disease. Neuropathol. Appl. Neurobiol. 2011; 37: 791–802. 21696416 10.1111/j.1365-2990.2011.01194.x

[11] Hoyte CO , Leikin JB . Management of diethylene glycol ingestion. Clin. Toxicol. (Phila.) 2012; 50: 525–7. 22694101 10.3109/15563650.2012.696199

